# A Cu-atom-chain current channel with a width of approximately 0.246 nm on (5, 0) single-wall carbon nanotube

**DOI:** 10.1038/s41598-017-13286-3

**Published:** 2017-10-10

**Authors:** Yue Wang, Kaigui Zhu, Qingyi Shao

**Affiliations:** 1 Laboratory of Quantum Engineering and Quantum Materials, School of Physics and Telecommunication Engineering, South China Normal University, Out Ring Road No. 378 Guangzhou University Town, Guangzhou, 510006 China; 2 Department of physics, Beihang University, Beijing, 100191 China; 3 School of Science, Jiangnan University, Wuxi, Jiangsu 214122 China

## Abstract

Continuous miniaturization with improved performance has enabled the development of electronic devices. However, further shrinking of electronic circuits will push feature sizes (linewidths) firmly into the nanoscale. This can cause electronic devices built using current materials (silicon-based) and fabrication processes to not work as expected. Therefore, new materials or preparation technologies are needed for the further miniaturization of electron devices. Here, through theoretical simulation, we show that regular doping of a Cu-atom chain on a single-wall carbon nanotube (SWCNT) can be used to realize an atomic-scale current channel (Cu-atom-chain current channel) with a linewidth of approximately 0.246 nm. Moreover, the atomic-scale Cu-atom-chain current channel shows enhanced conductivity (lower power consumption) compared to a pristine SWCNT. Such a Cu-atom-chain current channel with an atomic-scale linewidth and its method of fabrication (regular doping) may be suitable for the preparation of nanoelectronic devices.

## Introduction

Over the past few decades, the physical size of silicon-based electron devices has shrunk (miniaturization), which has led to a higher integrated chip density and more powerful performance^1^. This has been the direction of development for most electronics. However, further reduction of the smallest feature size (linewidth) for the realization of future silicon-based electronic devices will impinge on fundamental limits for physical scaling. As the linewidth approaches the nanoscale, silicon-based electron devices built using current materials and processing technologies tend to not work as expected. Therefore, it is necessary to explore alternative materials and preparation technologies for the further miniaturization of electron devices but with improved performance. One promising solution is to fabricate the electronic components using nanoscale or molecular-materials^2–5^, with carbon nanotubes^6^ (CNTs) considered as one of the most ideal candidate materials^7–9^. To realize devices based on such nanomaterials, it is important to start with a study focused on the fabrication of the current channel on the nanoscale.

Recently, carbon-based nanomaterials have received wide attention and application due to their outstanding properties, including mechanical performance^10,11^, self-assembly of multipodal junctions with interesting magnetic properties^12^, energy storage performance^13^ and electrical properties^14,15^. In particular, CNTs have been the subject of intense experimental research that indicates it is an ideal candidate material for the preparation of next-generation electronic devices with a narrow current channel and enhanced performance^16–22^. However, not all the properties of CNTs can meet the material requirements for the preparation of next-generation electronic devices. Some modifications in the processing of CNTs (such as doping and adsorbents) are needed to realize the desired electronic properties. CNT-copper (CNT-Cu) composites have received considerable attention both in experiment and theory due to the large free-electron density of Cu. CNT-Cu composites have been fabricated successfully using various methods, including electrochemical synthesis^23^, thermal evaporation^24^, electrodeposition^25,26^ and powder metallurgy^27^. These experimental studies have shown that CNT-Cu composites have high electrical conductivity^23,26^, excellent thermal conductivity^23,25^, a low coefficient of thermal expansion^23^ and improved photocurrent density (approximately 2.5 times compared to that of bare CNTs)^24^. In theory, the contact between the CNTs and a Cu matrix, and between CNTs and a Cu chain (Cu chain is perpendicular to the tube axis of the CNTs) has been investigated by Ghorbani-Asl^28^ and Kong^29^, respectively. The electronic properties of the CNT-Cu composites were found to be similar in both studies. Ghorbani-Asl found a scattering effect at the Cu-CNT interface, which can impede electron transport through the Cu-CNT composite. Kong’s results indicate that the contact region between a metallic CNT and Cu chain gives rise to a local energy gap (of approximately 0.1 eV) and charge depletion near the Fermi level, which can limit the electron transport between the metallic CNTs and Cu chain. The electronic properties of CNTs adsorbed onto a Cu chain have also been studied; these studies show that the electronic conductivity of CNTs can be enhanced by adsorbing a Cu chain on both the outside and inside walls of the CNT^30,31^. However, the electronic properties for a Cu-atom chain doped into a CNT have not been reported. In this work, we theoretically investigated the structure, stability and electron transport properties of a Cu-atom-chain doped (5, 0) SWCNT (Cu-chain-SWCNT). Our simulation results predict a Cu-chain-SWCNT with an atomic-scale current channel (width of 0.246 nm) on the Cu-atom-chain with enhanced conductivity compared to a pristine SWCNT.

## Results

### Structure and stability

**Figure 1:**
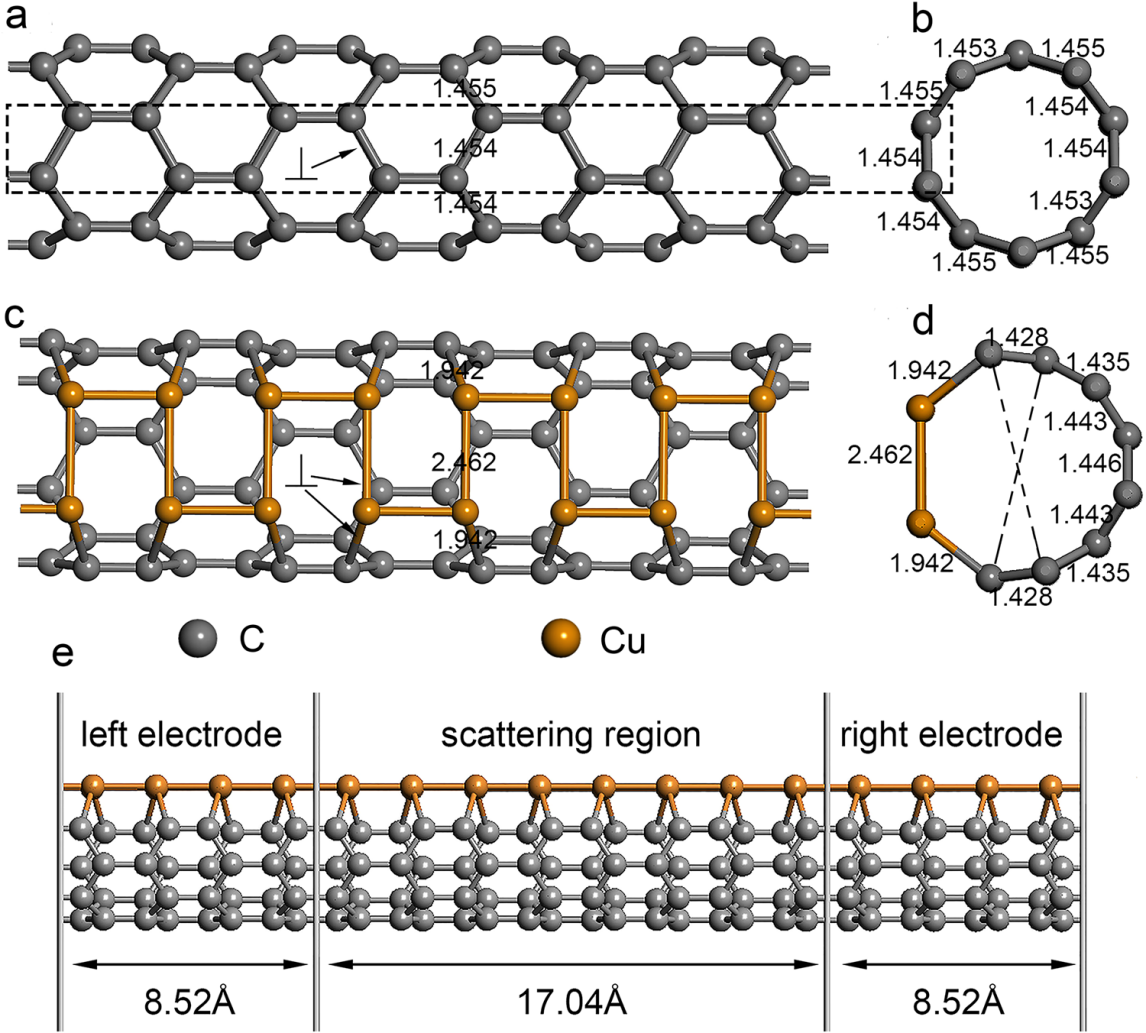
The geometrical structure of the pristine SWCNT and Cu-chain-SWCNT. (**a**) Top view of the pristine SWCNT; (**b**) cross section of the pristine SWCNT; (**c**) top view of the Cu-chain-SWCNT after geometry optimization; (**d**) cross section of the Cu-chain-SWCNT after geometry optimization; (**e**) the two-probe model used to calculate the transport properties of the Cu-chain-SWCNT, which includes three parts: left electrode, right electrode and scattering region of length 1.704 nm. The C atoms of the pristine SWCNT (black-dashed rectangle) show the doped position of the Cu-atom-chain. ⊥ denotes the bonds perpendicular to the tube axis in the pristine SWCNT and Cu-chain-SWCNT. The numbers in (**a**) to (**d**) show the bond lengths; the bond length unit is Å. The two black-dashed-lines in (**d**) stand for the diameter of Cu-chain-SWCNT.

Computational simulation of the pristine SWCNT and Cu-chain-SWCNT was based on density functional theory (DFT) and non-equilibrium Green function (NEGF) calculation. A metallic (5,0) SWCNT^32^ with a diameter of approximately 4 Å, which has been successfully prepared experimentally^33,34^, was chosen as the model basis. As shown in Fig. 1a (top view of the pristine SWCNT) and 1b (cross section of the pristine SWCNT) by the black-dashed-rectangle, the Cu-chain-SWCNT is formed by substitution of a section of the carbon-atom-chain in the pristine SWCNT by a Cu-atom-chain. The structure of the Cu-chain-SWCNT after geometry optimization is shown in Fig. 1c (top view) and 1d (cross section). First, the structure and stability of the Cu-chain-SWCNT after geometry optimization were examined. In Fig. 1a and c, the ⊥ symbol is used to identify the bonds in the pristine SWCNT and Cu-chain-SWCNT that are perpendicular to the tube axis. In Fig. 1a to d, the bond lengths (Å) for the pristine SWCNT and Cu-chain-SWCNT are also indicated by a number close to or on the bond. The two dashed lines in Fig. 1d indicate the tube diameter of Cu-chain-SWCNT.

There are some obvious differences between the structures of the Cu-chain-SWCNT and pristine SWCNT: (1) the angles are decreased from 120° between the ⊥C-C bonds and the tube axis to 90° between the ⊥Cu-Cu bonds and the tube axis and to 102° between ⊥Cu-C bonds and the tube axis; (2) the bond lengths are increased from 1.454 Å for the ⊥C-C bonds in the pristine SWCNT to 2.462 Å for the ⊥Cu-Cu bonds in the Cu-chain-SWCNT, and from 1.454/1.455 Å for the ⊥C-C bonds in the pristine SWCNT to 1.942 Å for the ⊥Cu-C bonds in the Cu-chain-SWCNT; (3) for the Cu-chain-SWCNT, the length of the ⊥Cu-Cu bonds (2.462 Å) and ⊥Cu-C bonds (1.942 Å) are much longer than the ⊥C-C bonds (1.454/1.455 Å); (4) the changes detailed above in (1), (2) and (3) result in the transformation of the circular cross section of the pristine SWCNT into a non-circular cross section for the Cu-chain-SWCNT and increase the cross section of the Cu-chain-SWCNT compared to that of the pristine SWCNT. The changes described above are due to the larger diameter of the Cu atom (290 pm) compared to the C atom (182 pm).

The stability of the Cu-chain-SWCNT was evaluated by calculating the formation energy (*E*_*f*_), which is defined by the following equation^35,36^:

1 $${E}_{f}={E}_{dop}-\,\frac{n(C)-n(Cu)}{n(C)}{E}_{SWCNT}-n(Cu)\mu (Cu)$$

where E_dop_ and E_SWCNT_ is the total energy of the Cu-chain-SWCNT and pristine SWCNT, respectively; *μ*(Cu) is the chemical potential of a Cu atom, calculated from the isolated Cu atom (in a 20 × 20 × 20 Å lattice) using DMOL^3^ code^37^; n(C) and n(Cu) are the number of C atoms in the pristine SWCNT and Cu atoms in the Cu-chain-SWCNT, respectively. The stability of doped SWCNT can be evaluated using the formation energy; the more negative the formation energy, the more stable they should be^35,37–39^. The calculated *E*_*f*_ of the Cu-chain-SWCNT is −0.325 eV/atom, indicating that the Cu-chain-SWCNT is energetically favorable. The stability of the SWCNT with two Cu-atom-chains doped at diametrically opposite positions (i.e., the second Cu-atom-chain doped at the opposite position of the Cu-atom-chain shown in Fig. 1c and d) was also calculated, and the result indicates a stable entity with a negative formation energy of −0.583 eV/atom.

### Electron transmission pathways and transmission eigenchannels

**Figure 2:**
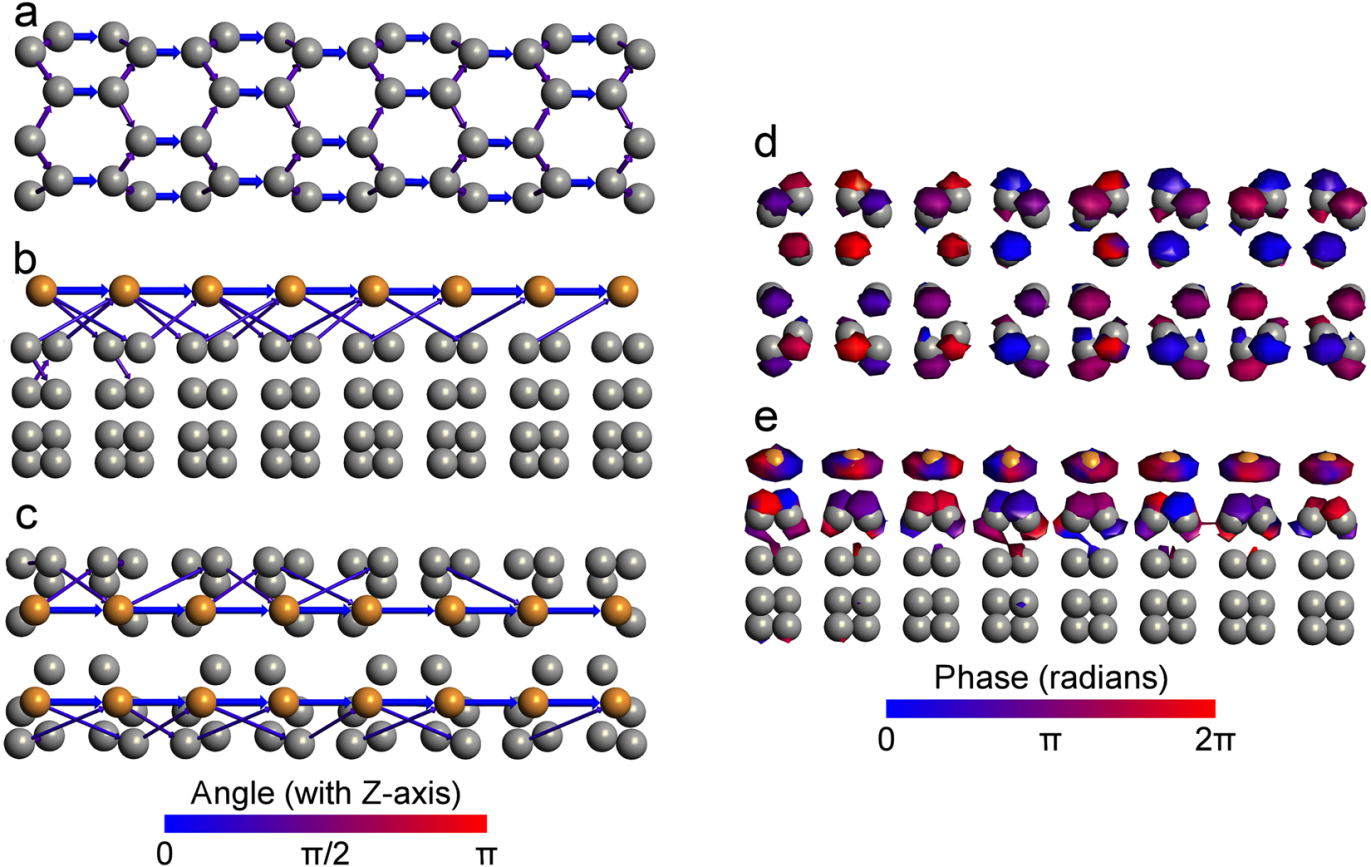
The electron transmission pathways and electron transmission eigenchannels under 1 eV voltage bias at the Fermi level. (**a**) Transmission pathways of the pristine SWCNT; (**b**) side view of the transmission pathways of the Cu-chain-SWCNT; (**c**) top view of transmission pathways of the Cu-chain-SWCNT; (**d**) electron transmission eigenchannels of the pristine SWCNT; (**e**) electron transmission eigenchannels of the Cu-chain-SWCNT. The thickness of the arrows indicates the level of the local current between each pair of atoms (a thicker arrow indicates a higher local current), and the colour of the arrows indicates the direction of the electron transport.

For the electron transport properties, we first investigated the electron transmission pathways of the pristine SWCNT (Fig. 2a) and Cu-chain-SWCNT (Fig. 2b and c). The transmission pathway can predict the pathway of electron transport through the pristine SWCNT and Cu-chain-SWCNT from the left to right electrodes under an external voltage bias^40^. Figure 2a shows that the electron transmission pathway of the pristine SWCNT is distributed evenly over the entire tube. However, it is considerably changed upon Cu-atom-chain doping into the SWCNT. As shown in Fig. 2b, the Cu-atom-chain dominates the electron transmission pathway, with little electron transmission occurring across the Cu-C bonds. Therefore, the Cu-atom-chain can be considered as the current channel (Cu-atom-chain current channel) on the Cu-chain-SWCNT. The width of Cu-atom-chain current channel can be defined by the distance between the two electron transport (current) pathways on Cu-atom-chain (see Fig. 2c), which is equal to the ⊥Cu-Cu bond length of 0.246 nm (see Fig. 1c). The width of the current channel on the Cu-atom-chain (0.246 nm) is smaller than the width of the current channel of the pristine SWCNT (a tube with a diameter of 0.4 nm). These findings can potentially provide a method to guide the behaviour of electron transport through control of the specific arrangement of doped atoms. In contrast, with conventional random doping, this type of doping, with a specific and well-defined arrangement of dopant atoms, can be called regular doping. The Cu-chain-SWCNT, with a unique, atomic-scale current channel, may be suitable in the fabrication of nanoelectron devices with very small dimensions and enhanced performance. For example, it is possible to create a p-n junction across the Cu-atom-chain current channel in the Cu-chain-SWCNT. Moreover, the transmission pathway for a two Cu-atom-chains doped SWCNT (doped at diametrically opposite positions) is found to be similar to that found for the pristine SWCNT, with an even distribution of the electron transmission over the entire tube. To study the influence of the length of the Cu-chain-SWCNT on the electron transport pathway, we increased the length of the scattering region from 1.704 nm to 3.408 nm. The simulation result indicates that the electron transport pathway of the Cu-chain-SWCNT (with a scattering region of 3.408 nm) is similar to the electron transport pathway found for the shorter Cu-chain-SWCNT shown in Fig. 2b and c.

To further understand the transport properties of the atomic-scale Cu-atom-chain current channel of the Cu-chain-SWCNT, we calculated the electron transmission eigenchannels, which are responsible for electron transport. Figure 2d indicates that the electron transmission eigenchannels of the pristine SWCNT are uniformly distributed throughout the entire tube. However, for the Cu-chain-SWCNT (see Fig. 2e), the transmission eigenchannels are mainly localized at the Cu-atom chain, with only a small fraction present at the C atoms connected to the Cu atoms. These results indicate that the electron transport of the Cu-chain-SWCNT is mainly controlled by the Cu-atom chain, in agreement with the results from the calculation of the electron transmission pathway.

### I-V characteristics and transmission spectra

**Figure 3:**
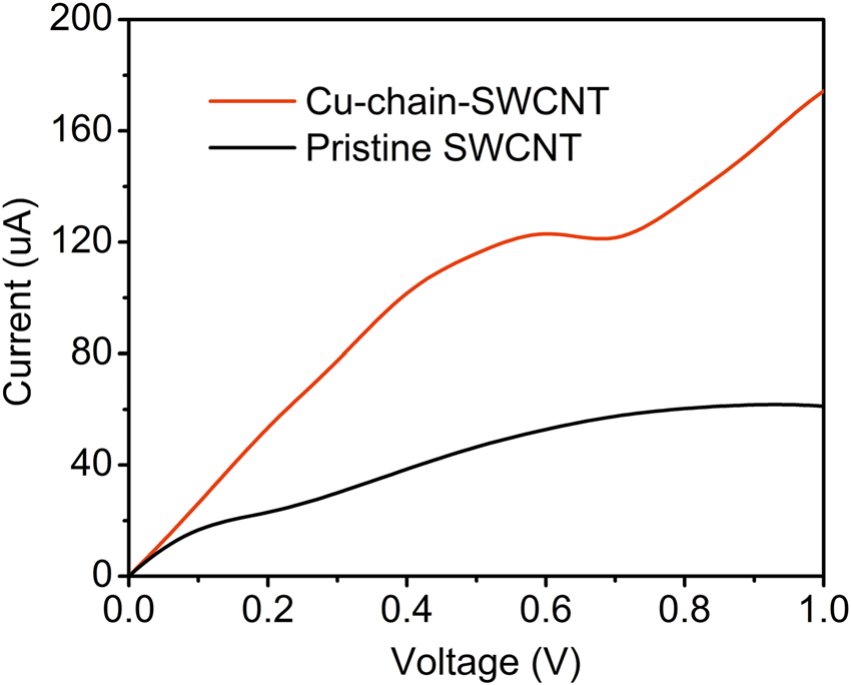
The I-V characteristics of the pristine SWCNT and Cu-chain-SWCNT.

The I-V properties of the pristine SWCNT and Cu-chain-SWCNT are shown in Fig. 3. The data show that the current in the Cu-chain-SWCNT is approximately 2–3 times higher than that in the pristine SWCNT at the same voltage (from 0.2 to 1 V). Therefore, the Cu-chain-SWCNT not only supports a very narrow atomic-scale current channel but also shows an increased conductance (smaller resistance) than the metallic pristine SWCNT. The current calculated in our work for the Cu-chain-SWCNT is higher than that found for B-N co-doped SWCNT^41^. The conductivity (σ) of the Cu-chain current channel (at a voltage of 1 V) was calculated using the equation:

2 $$\sigma \,=\,\frac{4IL}{VS}$$

where *V* (1 V) and *I* (0.174 mA) are the voltage and current, respectively; *S* (π*r* ^2^/*4*) and *L* (1.704 nm) are the cross-sectional area and length of the Cu-chain-SWCNT, respectively. The diameter of Cu-chain-SWCNT, *r* (0.47 nm), was calculated by taking the average value of the distances denoted by the two black-dashed-lines in Fig. 1d. The conductivity of Cu-chain-doped-SWCNT is calculated to be approximately 1.7 × 10^4^ S/cm, which is similar to the experimental value determined for a CNT-Cu composite (2.3–4.7 × 10^5^ S/cm)^26^. In equation (2), *S* should be the cross-sectional area of the Cu-atom-chain current channel (this value is not easy to evaluate). Consequently, the use of the cross-sectional area of Cu-chain-SWCNT gives an overestimate for *S*. Therefore, the conductivity of the Cu-chain current channel can be expected to be higher than 1.7 × 10^4^ S/cm. Figure 3 shows a nonlinear relationship between the current and voltage for both pristine SWCNT and Cu-chain-SWCNT, which indicates a non-Ohmic resistance.

**Figure 4:**
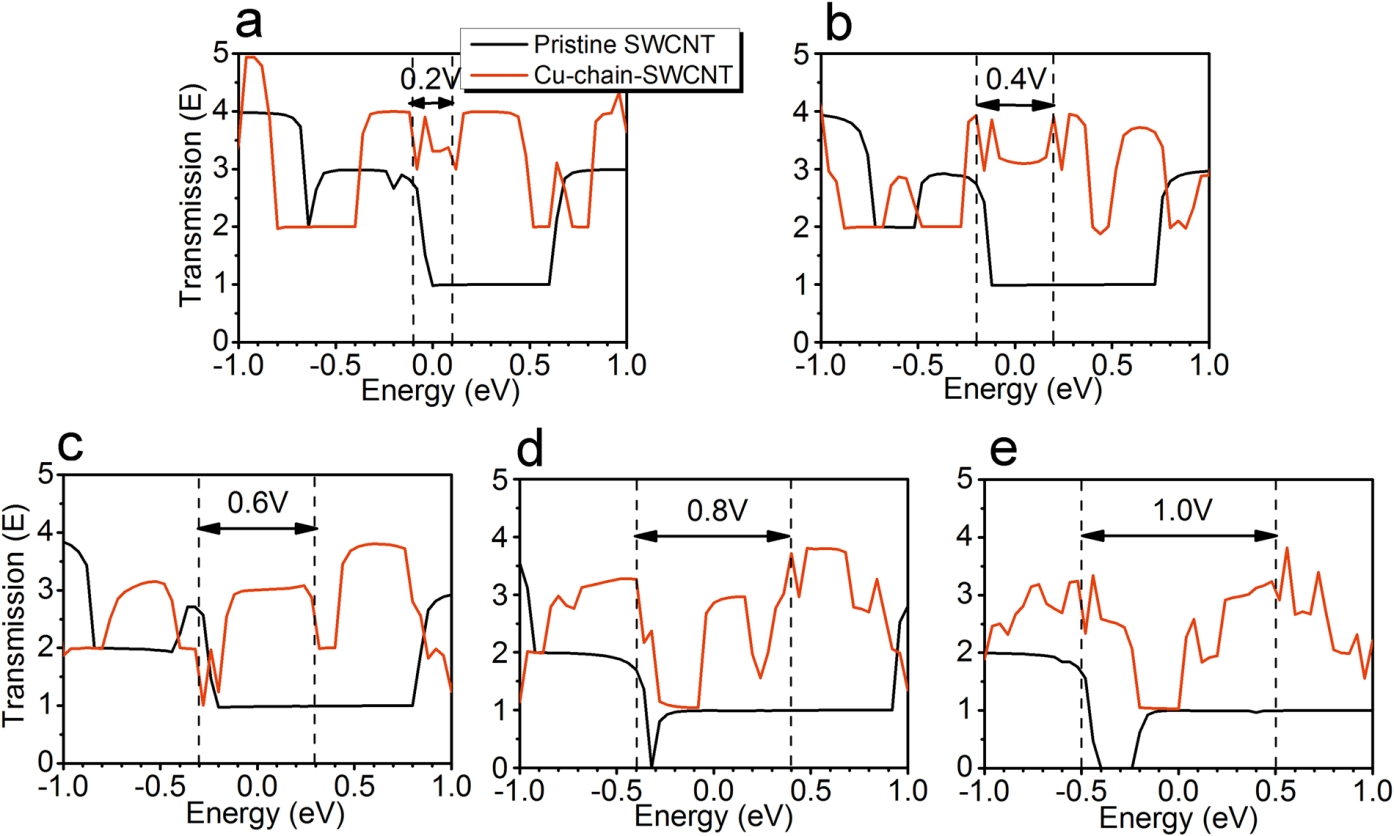
The transmission spectra of the pristine SWCNT and Cu-chain-SWCNT over a designated bias window. The black-dashed lines represent the bias window: (**a**) 0.2 V; (**b**) 0.4 V; (**c**) 0.6 V; (**d**) 0.8 V and (**e**) 1.0 V.

To gain a further understanding of the I-V characteristics, the transmission spectra of the pristine SWCNT and Cu-chain-SWCNT were calculated. A high value for the magnitude of the transmission, E, indicates a high conductance, and a high value for the integral of the transmission spectrum over the applied bias indicates a high current density. The transmission for the pristine SWCNT is approximately 1 at a bias window of 0.2, 0.4, 0.6, 0.8 and 1.0 V (see Fig. 4a to e). However, for the Cu-chain-SWCNT, E is approximately 3 at a bias window of 0.2, 0.4 and 0.6 V (see Fig. 4a to c) and approximately 2 at a bias window of 0.8 and 1.0 V (see Fig. 4d and e). These data imply higher conductivity and higher current density for the Cu-chain-SWCNT compared to the pristine SWCNT. Figure 4 also shows that E can vary non-monotonically with a changing voltage, resulting in a nonlinear resistance for the pristine SWCNT and Cu-chain-SWCNT.

### Band structures, DOS and Fermi levels

**Figure 5:**
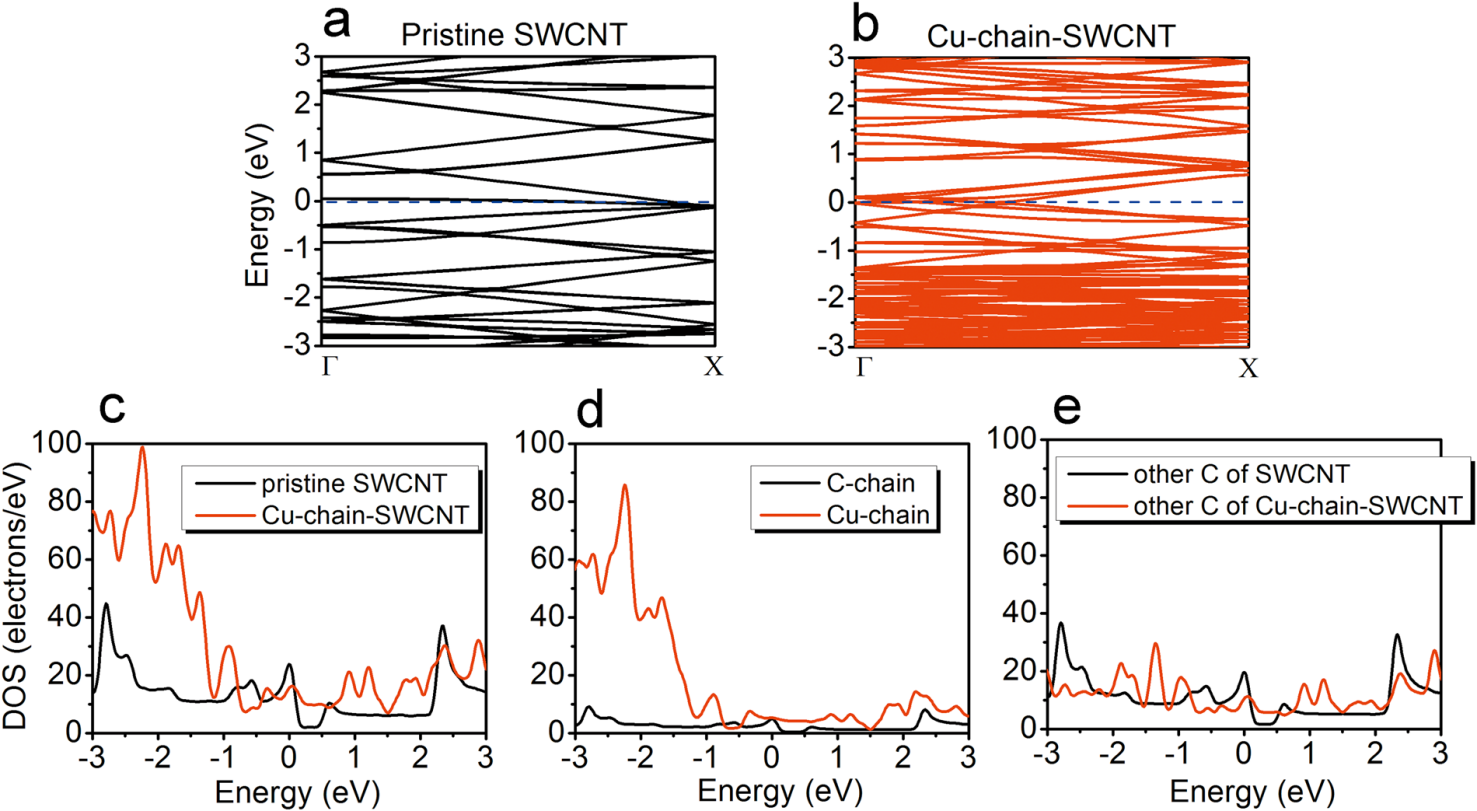
The band structure of the pristine SWCNT (**a**) and Cu-chain-SWCNT (**b**). The density of states of the pristine SWCNT and Cu-chain-SWCNT (**c**), the replaced C-atom-chain (C-chain) and doped Cu-atom-chain (Cu-chain) (**d**) and other C atoms of the pristine SWCNT (other C of SWCNT) and other C atoms of the Cu-chain-SWCNT (other C of Cu-chain-SWCNT) (**e**). The Fermi level was set at 0 eV for both the band structure and DOS. The term C-chain refers to the C-atom-chain shown by the black-dashed rectangle in Fig. 1a and b, with the other C atoms in Fig. 1a labelled as other C of SWCNT. The term Cu-chain refers to the Cu-atom-chain shown in Fig. 1c, with the other C atoms in Fig. 1c labelled as other C of Cu-chain-SWCNT.

To extend our understanding of the electronic properties of the pristine SWCNT and Cu-chain-SWCNT, the band structures, density of states (DOS) and positions of the Fermi levels were computed. As shown in Fig. 5a, b and c, compared with the pristine SWCNT, the Cu-chain-SWCNT show more energy bands and an increased DOS near the Fermi level. The Fermi level also increases from −5.599 eV for the pristine SWCNT to −5.203 eV for the Cu-chain-SWCNT; this increase in the Fermi level is as expected from a comparison of the band structure of the pristine SWCNT (see Fig. 5a) and Cu-chain-SWCNT (see Fig. 5b). The elevated Fermi level of the metallic Cu-chain-SWCNT indicates a higher free-electron concentration compared to the pristine SWCNT. The changes detailed above for the band structure, DOS and Fermi level imply that the conductivity of the pristine SWCNT can be enhanced by doping with a Cu-atom-chain and agrees with the calculated I-V characteristics and transmission spectral data. The results from our simulation are similar to those obtained by Yang *et al*.^30^, who investigated the transport properties of SWCNT with an adsorbed Cu-atom chain. They found enhanced conductivity for the SWCNT upon adsorption of a Cu-atom chain, which is also accompanied by an increase in the number of energy bands and DOS near the Fermi level and an upshift in the Fermi level. To further investigate the DOS of the Cu-chain-SWCNT, the projected density of states (PDOS) of the pristine SWCNT and Cu-chain-SWCNT were calculated. As shown in Fig. 5d and e, the calculations predict an increased DOS for the Cu-chain-SWCNT mainly due to the incorporation of the Cu-atom chain (see Fig. 5d); the PDOS of the other C on the Cu-chain-SWCNT and other C on the pristine SWCNT remain almost the same (see Fig. 5e). Recently, Ghorbani-Asl *et al*.^28^ studied the transport properties of Cu-CNT composites in which CNTs were fully embedded into a three-dimensional copper matrix and found that the Cu-CNT interface can lead to an electron scattering effect, which decreases the transmission through the Cu-CNT composite. However, the DOS of the Cu-CNT composite near the Fermi level shows a remarkable increase when compared with CNT, which is mainly contributed by the Cu atom. This is similar to our results, which show that the Cu-chain-SWCNT exhibits an increased DOS near the Fermi level compared with the SWCNT and that the increased DOS is mainly contributed by the Cu-atom chain; the DOS of the SWCNT near the Fermi level can be improved by substitutional doping of the Cu atom.

## Discussion

In summary, we used density functional theory and non-equilibrium Green’s function to study the structure, stability and electron transport properties of the pristine SWCNT and Cu-chain-SWCNT. Our calculations indicate that the Cu-chain-SWCNT is energetically stable despite possessing a slightly distorted tubular structure. Simulations for both the electron transmission pathway and transmission eigenchannel predict that an atomic-scale Cu-atom-chain current channel (with a width of 0.246 nm) is formed on the Cu-chain-SWCNT. The I-V characteristics and transmission spectral data indicate that both SWCNT and Cu-chain-SWCNT are non-Ohmic resistance, and the Cu-chain-SWCNT (or Cu-atom-chain current channel) exhibits enhanced conductivity compared to the pristine SWCNT. This enhancement is driven by an increase in the energy band number and DOS near the Fermi level and a raise of the Fermi level. These results highlight a potentially promising method of regular doping (such as atom-chain doping) to build atomic-scale current channel on SWCNT; this method may be applicable to other materials and suitable for the fabrication of nanoelectron devices.

## Methods

### Density functional theory

The calculations of the optimized geometry, stability, band structure, DOS and Fermi level of the pristine SWCNT and Cu-chain-SWCNT were carried out using DMOL^3^ code^42,43^ based on density functional theory (DFT). The structures of the SWCNT and Cu-chain-SWCNT were constructed using the Materials Studio program, which contains DMOL^3^ code. In this code, the electronic wave function was expanded in a double-numeric polarized (DNP) bias set with an orbital of 5.0 Å. For the exchange and correlation term, the generalized gradient approximation (GGA) was used as proposed by Perdew, Burke, and Ernzerhof (PBE)^44^. Compared with the local density approximation (LDA), the GGA shows several advantages and can provide more reliable results for the calculation of the electronic properties^38,45,46^. Geometry optimization was performed using a self-consistent field (SCF) convergence criterion of 1.0 × 10^−6^ Ha (1 Ha = 27.2114 eV), a maximum force of 0.002 Ha/Å and a maximum displacement of 0.005 Å.

### Atomistic-ToolKit

All the electronic transport properties of the Cu-chain-SWCNT were calculated using the framework provided by density functional theory (DFT) combined with the non-equilibrium Green’s function method (NEGF) and self-consistent extended Hückel theory (EHT)^47^, as implemented in the Atomistic-ToolKit (ATK) code^48,49^. The calculation was performed using a two-probe model (see Fig. 1e), which has been previously used to study the electron transport properties^41,50,51^. As shown in Fig. 1e, the two-probe model contains three parts: left electrode, central region and right electrode. The current (*I*) that passes through the central region under an applied voltage (*V*) was calculated by the Landauer-Buttiker formula^52^. The current is given by equation:

3 $$I({V}_{b})=\frac{2e}{h}\int T(E,{V}_{b})[f(E-{\mu }_{L})-f(E-{\mu }_{R})]dE$$

where *T*(*E, V*_*b*_) is the transmission coefficient of the device for an electron at energy *E* with a voltage bias *V*_*b*_. *f* (*E* − *μ*_*L/R*_) is the Fermi distribution for the left/right electrodes. *μ*_*L/R*_ = *E*_*F*_ *± V*_*b*_/2 is the chemical potential of the left/right electrodes, *E*_*F*_ is the Fermi energy of the device.
